# Interleukin‐23 Receptor and Interleukin‐17 Receptor A: Splice Variants, Isoforms and Their Relationship With Periodontitis—A Systematic Review and Bioinformatic Analysis

**DOI:** 10.1111/jcmm.71325

**Published:** 2026-08-17

**Authors:** Mario Alberto Alarcón‐Sánchez, Celia Guerrero‐Velázquez, Daniel Ortuño‐Sahagún, Sarah Monserrat Lomelí‐Martínez, Julieta Sarai Becerra‐Ruiz, Javier Flores‐Fraile, Artak Heboyan

**Affiliations:** ^1^ Molecular Biology in Medicine Program, University Center of Health Sciences University of Guadalajara (CUCS‐UdeG) Guadalajara Jalisco Mexico; ^2^ Institute of Research in Dentistry, Department of Integral Dental Clinics, University Center of Health Sciences University of Guadalajara (CUCS‐UdeG) Guadalajara Jalisco Mexico; ^3^ Laboratory of Molecular Neuroimmunobiology, Institute of Translational Neurosciences, Department of Molecular Biology and Genomics, University Center for Health Sciences University of Guadalajara (CUCS‐UdeG) Guadalajara Jalisco Mexico; ^4^ Department of Medical and Life Sciences, La Ciénega University Center University of Guadalajara (CUCIENEGA‐UdeG) Ocotlán Jalisco Mexico; ^5^ Department of Clinics, Los Altos University Center University of Guadalajara (CUALTOS‐UdeG) Tepatitlán de Morelos Jalisco Mexico; ^6^ Department of Surgery, Faculty of Medicine University of Salamanca Salamanca Spain; ^7^ Department of Research Analytics, Saveetha Dental College and Hospitals, Saveetha Institute of Medical and Technical Sciences Saveetha University Chennai India; ^8^ Department of Prosthodontics, Faculty of Stomatology Yerevan State Medical University after Mkhitar Heratsi Yerevan Armenia; ^9^ Department of Prosthodontics, School of Dentistry Tehran University of Medical Sciences Tehran Iran

**Keywords:** Interleukin‐17 Receptor A, Interleukin‐23 Receptor, isoforms, periodontitis, splicing variants

## Abstract

This systematic review aimed to: (1) identify the splicing variants of *IL23R* and *IL17RA* reported in the literature; (2) perform a multiple alignment analysis to describe the isoforms of IL‐23R and IL‐17RA; and (3) compare the expression levels of IL‐23R, IL‐17RA, and their soluble isoforms (sIL‐23R and sIL‐17RA) in patients with periodontitis and periodontally healthy individuals. The study protocol followed PRISMA guidelines and was registered in PROSPERO (CRD420251267367). Six databases (PubMed, ScienceDirect, Scopus, Web of Science, EBSCO, and Google Scholar) were searched without restrictions on year or language. The descriptors used were: ‘Interleukin‐23 Receptor,’ ‘IL‐23R,’ ‘Interleukin‐17 Receptor A’ ‘IL‐17RA,’ ‘Alternative Splicing,’ ‘Splice Variants,’ ‘Isoforms,’ and ‘Periodontitis.’ The bioinformatics analysis was performed using CLUSTALW (V.1.83), InterPro and DeepTMHMM. Risk of bias was assessed with the QUIN and JBI tools for cross‐sectional studies. Of 104 articles, four in vitro studies and eight cross‐sectional studies were included. Qualitative analysis revealed that to date there are 32 splicing variants of the *IL23R* gene, while only one splicing variant has been reported for *IL17RA*. CLUSTALW, InterPro and DeepTMHMM analysis showed that these splicing variants result in 23 isoforms which can be soluble forms, complete intracellular peptides, truncated extracellular or intracellular peptides, or complete structures with truncated extracellular and/or intracellular domains. All studies had a low risk of bias. IL‐23R and IL‐17RA exhibit structural diversity resulting from alternative splicing, with IL‐23R demonstrating significantly greater isoform complexity. However, the biological significance of these isoforms in periodontitis remains unclear and requires further investigation.

**Abbreviations**
GM‐CSF: granulocyte‐macrophage colony‐stimulating factor
IFN‐γ: interferon gamma
IL‐17: interleukin 17
IL‐17RA: interleukin 17 receptor A
IL‐1β: interleukin 1 beta
IL‐21: interleukin 21
IL‐22: interleukin 22
IL‐23: interleukin 23
IL‐23R: interleukin 23 receptor
IL‐6: interleukin 6
IL‐8: interleukin 8
JAK: Janus kinase
RANK: receptor activator of nuclear factor κB
RANKL: receptor activator of nuclear factor κB ligand
STAT: signal transducer and activator of transcription
TAK1: TGF‐beta‐activated kinase 1
TGF‐β: transforming growth factor beta
TNF‐α: tumour necrosis factor alpha
TRAF6: TNF receptor‐associated factor 6

## 1 Introduction

Periodontitis is a chronic, destructive inflammatory disease of the tissues that support the teeth [1]. The persistent challenge of a polymicrobial dysbiotic biofilm dominated by anaerobic and facultative anaerobic gram‐negative bacteria, such as *Veillonella parvula* , *Campylobacter gracilis* , *Prevotella* spp., *Aggregatibacter actinomycetemcomitans* , *Tannerella forsythia* , *Treponema denticola* , and *Porphyromonas gingivalis* [2], along with other modifiable risk factors (smoking, poor hygiene, stress, alcohol consumption, nutritional and psychosocial deficiencies) and non‐modifiable factors such as age, genetic predisposition, and systemic diseases, is the main cause of the development and progression of the disease [3]. Its prevalence is 62.3%, and in its most severe form, it can affect up to 23.6% of the world's population [4], making it a persisting public health problem with functional, aesthetic, and economic repercussions [5].

Periodontitis is characterized by an exacerbated immunoinflammatory response mediated by cytokines, among which the interleukin‐23/interleukin‐17 (IL‐23/IL‐17) axis and its receptors (IL‐23R/IL‐17RA) play a central role [6]. The pathogen‐associated molecular patterns of the main periodontopathogenic species of the Socransky complex ( *P. gingivalis* , *T. forsythia* , and *T. denticola* ) activate the pattern recognition receptors of gingival keratinocytes, stimulating the production of proinflammatory cytokines such as interleukin‐1 beta (IL‐1β), IL‐8, and tumour necrosis factor alpha (TNF‐α), which contribute to the recruitment of macrophages, dendritic cells, neutrophils, and natural killer cells [7]. Dendritic cells capture antigens and migrate from the site of inflammation and destruction to the lymph nodes, where they present the antigen to naive CD4+ T cells. This interaction, together with a specific cytokine profile, contributes to the differentiation of these cells into different subsets. In particular, transforming growth factor beta (TGF‐β), IL‐1β, IL‐6, TNF, and IL‐21 are necessary for the differentiation of Th17 cells [8].

In addition, dendritic cells and macrophages secrete proinflammatory cytokines such as IL‐23. IL‐23 binds to its receptor (IL‐23R) on the membrane of Th17 cells, maintaining clonal expansion and increasing the levels of other proinflammatory cytokines, including TNF‐α, IL‐6, IL‐21, IL‐22, granulocyte‐macrophage colony‐stimulating factor (GM‐CSF), interferon gamma (IFN‐γ), receptor activator of nuclear factor κB ligand (RANKL), and IL‐17. IL‐17A binds to its receptor (IL‐17RA) on the membrane of osteoblasts, fibroblasts, and periodontal ligament cells, increasing RANKL expression. RANKL then binds to its receptor (RANK) on the membrane of osteoclast precursor cells, promoting their differentiation (osteoclastogenesis) into active osteoclasts and inducing alveolar bone loss, which is characteristic of periodontitis [9, 10].

Research on the receptors in this pathway has revealed greater structural and functional complexity than initially recognized. Both IL‐23R and IL‐17RA are members of the Type I cytokine receptor family, characterized by extracellular domains rich in immunoglobulin and fibronectin motifs, as well as intracellular signalling domains dependent on TRAF6/TAK1 or JAK/STAT kinases [11, 12]. However, beyond their canonical forms, both genes undergo alternative splicing events that generate diverse isoforms with potential modulatory functions [13, 14, 15, 16] The identification and characterization of splicing variants and isoforms of the IL‐23R and IL‐17RA receptors in periodontitis is an emerging field with important biomedical implications. If certain isoforms act as negative regulators of IL‐23/IL‐17 axis signalling, they could serve as diagnostic or prognostic biomarkers and as potential therapeutic targets for specific biological strategies, as has already been achieved with IL‐17RA antagonists in plaque psoriasis [17]. However, to date, no systematic review has integrated and critically analysed the available evidence on splicing variants, isoforms, and their possible biological roles in periodontitis. Therefore, this systematic review aimed to: (1) identify the splicing variants of *IL23R* and *IL17RA* reported in the literature; (2) perform bioinformatic analysis to describe the isoforms of IL‐23R and IL‐17RA; and (3) compare the expression levels of IL‐23R, IL‐17RA, and their soluble isoforms (sIL‐23R and sIL‐17RA) in patients with periodontitis and periodontally healthy individuals.

## 2 Materials and Methods

### 2.1 Reporting Format

The study protocol was reported according to PRISMA guidelines [18].

### 2.2 PICOT/PECOS System

- *Population*: (1) Cell lines from various types of human and murine cancer, peripheral blood mononuclear cells from healthy subjects, and systemically healthy adults.
- *Intervention*/*exposure*: Stimulation with mitogens (concanavalin A, lipopolysaccharides, phytohemagglutinin; phorbol myristate acetate plus ionomycin); patients with periodontitis.
- *Comparison*: Healthy lung tissue; periodontally healthy individuals.
- *Outcomes*: Expression of *IL23R* and *IL17RA* splicing variants and description of possible isoforms; expression levels of IL‐23R and IL‐17RA and their soluble isoforms (sIL‐23R and sIL‐17RA).
- *Type of studies*: Experimental in vitro studies with a cross‐sectional design.

### 2.3 Research Questions

1. What splicing variants of *IL23R* and *IL17RA* are reported in the literature?
2. What possible isoforms are generated from these variants?
3. Are there differences in the expression levels of IL‐23R, IL‐17RA, and their soluble isoforms (sIL‐23R and sIL‐17RA) in patients with periodontitis compared to periodontally healthy individuals?

### 2.4 Criteria for Selection

The inclusion criteria were in vitro experimental studies evaluating the expression of splicing variants of *IL23R* and *IL17RA* in cell lines from various types of cancer, such as human squamous laryngeal carcinoma (Hep2), human lung carcinoma (A549), human chronic myeloid leukaemia (K62), human gastric adenocarcinoma (SGC‐7901), human hepatoma (HepG2), human pancreatic carcinoma (CAP1), and human breast cancer (CAM). Additional cell lines included were the human embryonic kidney cell line (293 T), human submandibular gland cell line (A‐253), human bronchial cells (BEAS‐2B), and peripheral blood mononuclear cells from healthy subjects. Cross‐sectional clinical studies were also included if they evaluated the expression levels of IL‐23R and IL‐17RA and their soluble isoforms in biological samples such as serum, plasma, and gingival tissue from patients over 35 years of age and both sexes, using techniques such as PCR, ELISA, immunofluorescence, and Western blot. Subjects with any systemic disease, smokers, alcohol consumers, those undergoing orthodontic treatment, pregnant women, and patients receiving antibiotics or anti‐inflammatory drugs were excluded. Randomized controlled trials, cohort studies, animal model studies, conference abstracts, theses, bibliometric analyses, reviews, and meta‐analyses were also excluded. No language or publication year restrictions were applied.

### 2.5 Information Sources

M.A.A.‐S. and A.H. independently searched six electronic databases: PubMed, Scopus, ScienceDirect, Web of Science, Dentistry & Oral Science Source, and Google Scholar. They used combinations of the following MeSH terms: ‘Interleukin‐23 Receptor,’ ‘IL‐23R,’ ‘Interleukin‐17 Receptor A,’ ‘IL‐17RA,’ ‘Alternative Splicing,’ ‘Splice Variants,’ ‘Isoforms,’ ‘Periodontitis’ along with the Boolean operators ‘OR’ and ‘AND’. The search was conducted through December 4, 2025.

### 2.6 Search Strategy

For PubMed, the search strategy was: (((((((Interleukin‐23 Receptor) OR (IL‐23R)) OR (Interleukin‐17 Receptor A)) OR (IL‐17RA)) AND (Alternative Splicing)) OR (Splice Variants)) AND (Isoforms)) AND (Periodontitis), yielding 5 papers. For Scopus, the search was: TITLE‐ABS‐KEY (‘Interleukin‐23 Receptor’ OR ‘IL‐23R’ OR ‘Interleukin‐17 Receptor A’ OR ‘IL‐17RA’) AND TITLE‐ABS‐KEY (‘Alternative Splicing’ OR ‘Splice Variants’) AND TITLE‐ABS‐KEY (‘Isoforms’) AND TITLE‐ABS‐KEY (‘Periodontitis’), yielding 6 papers. For ScienceDirect, the search was: (‘Interleukin‐23 Receptor’ OR ‘IL‐23R’ OR ‘Interleukin‐17 Receptor A’ OR ‘IL‐17RA’) AND (‘Alternative Splicing’ OR ‘Splice Variants’) AND (‘Isoforms’) AND (‘Periodontitis’), yielding 9 papers. For Web of Science, the search was: (((((((ALL = (Interleukin‐23 Receptor)) OR ALL = (IL‐23R)) OR ALL = (Interleukin‐17 Receptor A)) OR ALL = (IL‐17RA)) AND ALL = (Alternative Splicing)) OR ALL = (Splice Variants)) AND ALL = (Isoforms)) AND ALL = (Periodontitis), yielding 1 result. For Dentistry & Oral Science Source, the search was: (((((((Interleukin‐23 Receptor) OR (IL‐23R)) OR (Interleukin‐17 Receptor A)) OR (IL‐17RA)) AND (Alternative Splicing)) OR (Splice Variants)) AND (Isoforms)) AND (Periodontitis), yielding 1 result. For Google Scholar, the search was: (((((((Interleukin‐23 Receptor) OR (IL‐23R)) OR (Interleukin‐17 Receptor A)) OR (IL‐17RA)) AND (Alternative Splicing)) OR (Splice Variants)) AND (Isoforms)) AND (Periodontitis), yielding 80 papers.

### 2.7 Study Selection

References were transferred in RIS format to the Rayyan platform (www.rayyan.ai) [19] accessed on December 10, 2025, where the study selection process was conducted. Duplicate articles were removed first, followed by screening of titles and abstracts. Next, and in accordance with the eligibility criteria, the full texts of the remaining studies were examined to determine inclusion. This phase was conducted out independently by two researchers (M.A.A.‐S. and S.M.L.‐M.). Discrepancies were resolved by consensus.

### 2.8 Data Extraction

Data extraction was conducted independently by two researchers (M.A.A.‐S. and A.H.). The variables considered were the first author's name and year of publication, the country where the research was conducted, study design, name of the splicing variant and isoform, type of protein translated, sex and age of the patients, number of cases (patients with periodontitis) and controls (periodontally healthy individuals), total study population, type of receptor evaluated, biological sample, technique used, and clinical results. Differences were resolved through discussion with the rest of the team.

### 2.9 Outcomes

The primary outcomes were the expression of splicing variants of *IL23R* and *IL17RA*, and the description of possible isoforms using bioinformatic analysis. A splicing variant is defined as a different version of mRNA generated from the same gene due to modifications in the alternative splicing process. These transcripts produce different proteins (isoforms) with similar structures but different functions [20]. Secondary outcomes included comparison of the expression levels of IL‐23R and IL‐17RA and their soluble isoforms (sIL‐23R and sIL‐17RA) in patients with periodontitis and periodontally healthy individuals.

### 2.10 Quantitative Synthesis

Quantitative meta‐analysis was not feasible due to heterogeneity in biological samples (serum, plasma, gingival tissue), analytical techniques (PCR, ELISA, immunofluorescence, Western blot), periodontal phenotype, and outcome reporting formats, which precluded standardized effect size calculation.

### 2.11 Bioinformatic Analysis

#### 2.11.1 Multiple Alignment Analysis

Multiple sequence alignment analysis was performed using CLUSTALW (www.genome.jp/tools‐bin/clustalw) [21]. This bioinformatics software compares multiple DNA, RNA, or protein sequences to identify similarities at the nucleotide or amino acid level. All FASTA sequences available in GenBank (www.ncbi.nlm.nih.gov/genbank/) [22] for proteins translated from the splicing variants of both receptors (IL‐23R and IL‐17RA) were downloaded. Each amino acid sequence of the isoforms was then aligned with the canonical sequence of the receptors. The UNIPROT platform (www.uniprot.org) [23] was consulted to determine the positions of the signal peptide, extracellular domain, transmembrane domain, and intracellular domain of the proteins.

#### 2.11.2 InterPro Analysis

Functional domains and sites were predicted using the InterPro platform (www.ebi.ac.uk/interpro/) [24].

#### 2.11.3 DeepTMHMM Analysis

Transmembrane regions were identified using the DeepTMHMM deep learning algorithm (www.dtu.biolib.com/DeepTMHMM) [25].

### 2.12 Bias Analysis of Included Studies

The methodological quality of the in vitro studies was assessed using the QUIN tool [26] This system evaluates 12 items: clarity of objectives, sample size calculation, sampling technique, details of the comparison group, methodology, details of the operator, randomization, measurement of results, details of the evaluator of the results, blinding, statistical analysis, and presentation of results. Scores were assigned as follows: (I) adequately specified = 2; (II) inadequately specified = 1; (III) not specified = 0; and (IV) not applicable. Articles with scores above 70% were considered to have low bias; 50%–70% moderate bias; and below 50% high bias.

The quality of cross‐sectional studies was assessed using the Joanna Briggs Institute (JBI) tool [27] This system evaluates eight items: inclusion criteria, description of study subjects, measurement of exposure, measurement of the condition, identification of confounding factors, strategies to address confounding factors, measurement of outcomes, and statistical analysis. Scores were represented as yes, no, unclear, or not applicable. Articles with scores above 70% were considered to have low bias; 40%–60% moderate bias; and below 30% high bias.

### 2.13 PROSPERO Register

The study protocol was registered prior to data extraction and risk of bias assessment (December 13, 2025), with access number: CRD420251267367.

## 3 Results

### 3.1 Study Selection

Initially, 104 articles were identified in the following databases:

- PubMed (5 articles).
- ScienceDirect (9 articles).
- Scopus (6 articles).
- Web of Science (3 articles).
- EBSCO (1 article).
- Google Scholar (80 articles).

**FIGURE 1:**
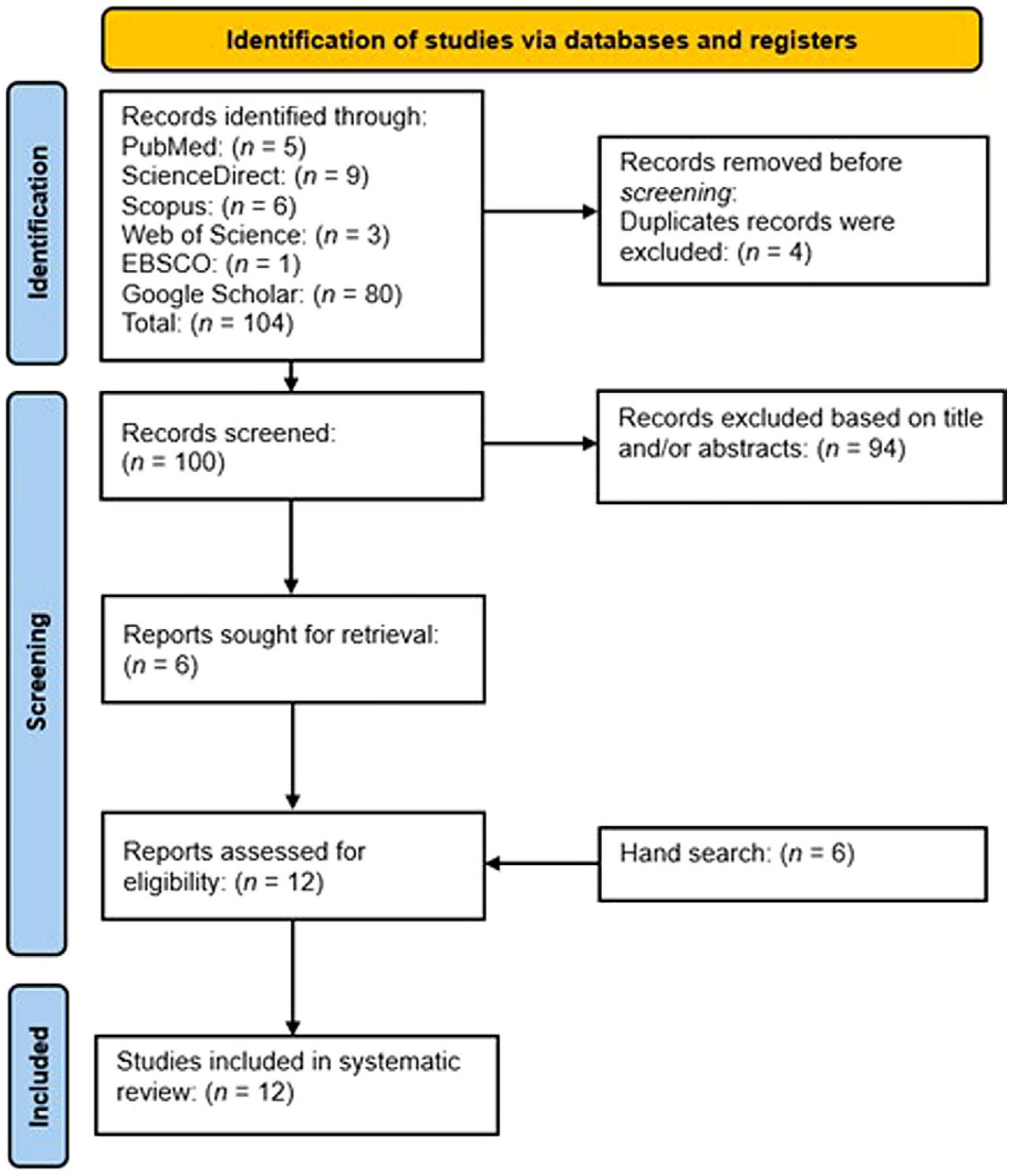
PRISMA flow diagram. PRISMA, Preferred Reporting Items for Systematic and Meta‐Analyses.

Four duplicates were then discarded, leaving a total of 100 articles. In the screening phase, 94 studies were excluded based on the title and abstract, leaving a total of 6 potentially eligible articles. In addition, a manual search was performed, and another 6 articles were found. After reviewing the full text, no articles were excluded. Therefore, 12 studies were included in this systematic review (Figure 1) [13, 14, 15, 16, 28, 29, 30, 31, 32, 33, 34, 35].

### 3.2 *IL23R* and *IL17RA* Splicing Variants

To date, 32 splicing variants of the *IL23R* gene have been reported. In contrast, only one splicing variant has been reported for *IL‐17RA*. Exon deletions are listed in Table 1.

**TABLE 1:** Splicing variants and isoforms of IL‐23R and IL‐17RA receptors.

Author and year	Splicing variant	Isoform	Protein type translated	Same structure
	IL‐23R	Canonical protein	Complete structure	
Zhang et al. (2006)	IL‐23R (Δ5 y 7)^a^	Isoform 2 (F1)	Truncated extracellular peptide	
	IL‐23R (Δ5 y 7)^a^	Isoform 2 (F2)	Complete structure with truncated extracellular domain	
	IL‐23R (Δ7 y 10)^a^	Isoform 3 (F1)	Soluble form	3 y 4
	IL‐23R (Δ7 y 10)^a^	Isoform 3 (F3)	Intracellular peptide	
	IL‐23R (Δ7, 10 y Δ12 67 nt)	Isoform 4	Soluble form	3 y 4
	IL‐23R (Δ1‐6, 9 y 10)	Isoform 5	Truncated intracellular peptide	
	IL‐23R (Δ1‐6)	Isoform 6	Complete structure with truncated extracellular domain	
Mancini et al. (2008)	IL‐23R (9a/pΔ5 71 nt)	Isoform 7	Truncated extracellular peptide or truncated intracellular peptide	7, 19 y 29
Kan et al. (2008)	IL‐23R (Δ4)	Isoform 8	Truncated extracellular peptide	
	IL‐23R (Δ5)	Isoform 9	Truncated extracellular peptide or truncated intracellular peptide	9, 22 y 23
	IL‐23R (Δ6)	Isoform 10	Truncated extracellular peptide	
	IL‐23R (Δ7)	Isoform 11	Truncated extracellular peptide	
	IL‐23R (Δ8)	Isoform 12	Complete structure with truncated extracellular and intracellular domains	
	IL‐23R (Δ9)	Isoform 13	Truncated extracellular peptide	
	IL‐23R (Δ5 y 6)	Isoform 14	Truncated extracellular peptide or truncated intracellular peptide	14, 24 y 25
	IL‐23R (Δ6 y 7)	Isoform 15	Complete structure with truncated extracellular and intracellular domains	
	IL‐23R (Δ8 y 9)	Isoform 16	Truncated extracellular peptide	
	IL‐23R (Δ5, 6 y 7)	Isoform 17	Truncated extracellular peptide or truncated intracellular peptide	
	IL‐23R (pΔ5 5 nt)	Isoform 18	Truncated extracellular peptide or truncated intracellular peptide	18, 28 y 31
	IL‐23R (pΔ5 71 nt)	Isoform 19	Truncated extracellular peptide or truncated intracellular peptide	7, 19 y 29
	IL‐23R (pΔ11 67 nt)	Isoform 20	Complete structure with truncated extracellular and intracellular domains	
	IL‐23R (pΔ5, 6 y 7)	Isoform 21	Truncated extracellular peptide or truncated intracellular peptide	
	IL‐23R (Δ5 y 8)	Isoform 22	Truncated extracellular peptide or truncated intracellular peptide	9, 22 y 23
	IL‐23R (Δ5 y 9)	Isoform 23	Truncated extracellular peptide or truncated intracellular peptide	9, 22 y 23
	IL‐23R (Δ5, 6 y 8)	Isoform 24	Truncated extracellular peptide or truncated intracellular peptide	14, 24 y 25
	IL‐23R (Δ5, 6 y 9)	Isoform 25	Truncated extracellular peptide or truncated intracellular peptide	14, 24 y 25
	IL‐23R (pΔ7, 8; pΔ11 67 nt)	Isoform 26	Truncated extracellular peptide	
	IL‐23R (pΔ5, 6, 7; Δ9; pΔ11 67 nt)	Isoform 27	Truncated extracellular peptide	
	IL‐23R (pΔ5, 5 nt; Δ6 y 7)	Isoform 28	Truncated extracellular peptide or truncated intracellular peptide	18, 28 y 31
	IL‐23R (pΔ5, 71 nt; Δ8 y 9)	Isoform 29	Truncated extracellular peptide or truncated intracellular peptide	7, 19 y 29
	IL‐23R (Δ8, pΔ11 67 nt)	Isoform 30	Complete structure with truncated extracellular and intracellular domains	
	IL‐23R (pΔ5, 5 nt; Δ8)	Isoform 31	Truncated extracellular peptide or truncated intracellular peptide	18, 28 y 31
	IL‐17RA	Canonical protein	Complete structure	
Sohda et al., 2013	IL‐17RA (pΔ11)	Isoform 2	Soluble form	

^a^ The IL‐23R2 and IL‐23R3 splicing variants generate an alternative reading frame, giving rise to different proteins. Δ Complete deletion of the exon; pΔ Partial deletion of the exon.

### 3.3 In Silico Characterization of IL‐23R Isoforms

IL‐23R consists of 629 amino acids; the signal peptide spans positions 1–23, the ectodomain 24–355, the transmembrane domain 356–376, and the endodomain 377–629 (www.dtu.biolib.com/DeepTMHMM). Three key domains are identified in the ectodomain: the immunoglobulin D1 domain and two type 3 fibronectin domains (D2 and D3) [36].

**FIGURE 2:**
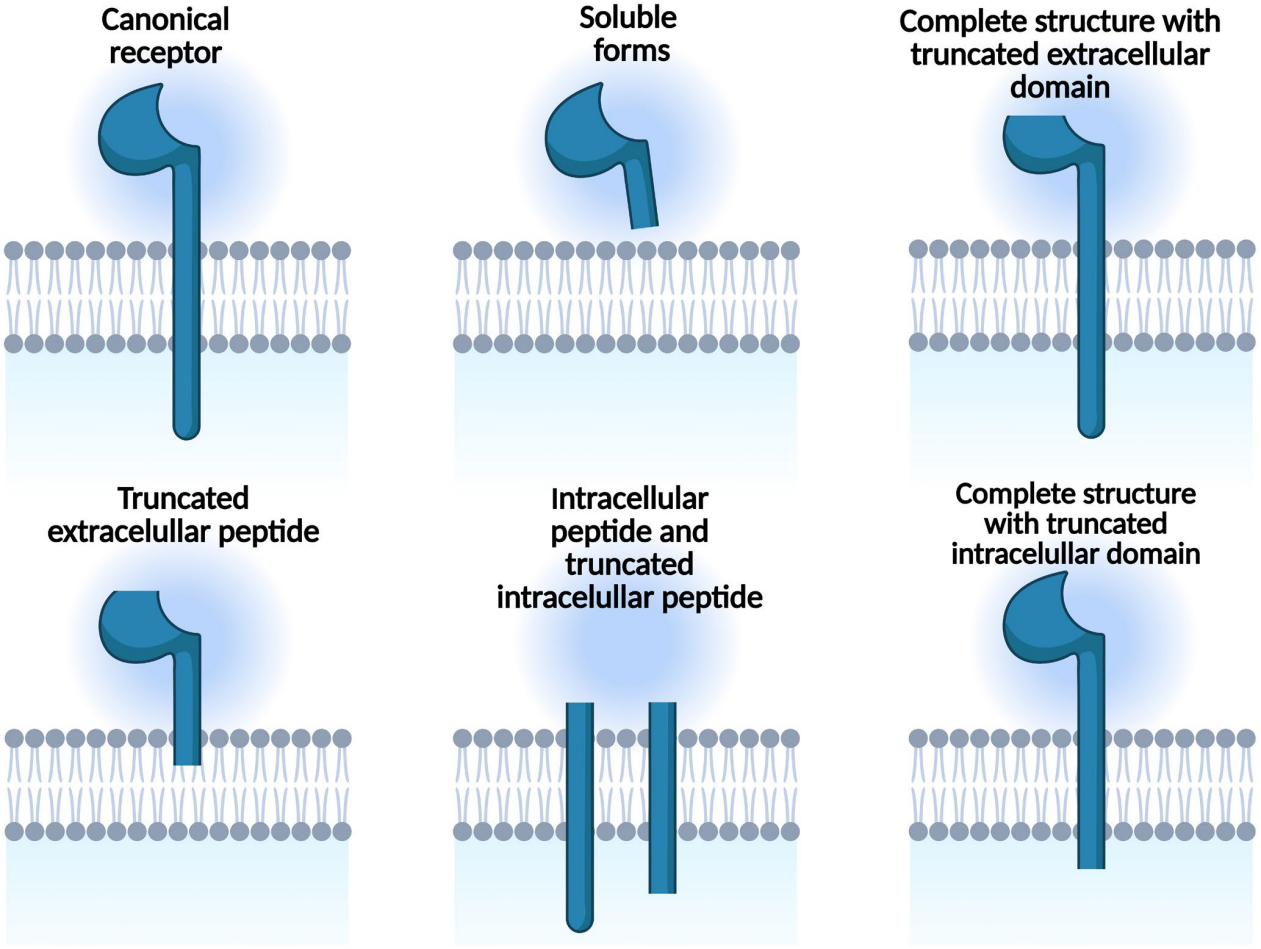
Predicted structural isoforms of IL‐23R and IL‐17RA based on multiple alignment analysis of reported splicing variants and available protein sequences. Some splicing variants were experimentally identified in previous studies [13, 14, 15, 16]; however, protein structures illustrated correspond to computationally predicted isoforms derived from bioinformatic alignment analysis. Created in https://BioRender.com.

Eight isoforms (2‐F1, 8, 10, 11, 13, 16, 26, and 27) correspond to truncated extracellular peptides (Figure 2). InterPro analysis revealed regions consistent with incomplete immunoglobulin‐like and Type III fibronectin domains, indicating partial loss of the D1–D3 extracellular domains. DeepTMHMM identified the absence of the transmembrane region and the endodomain (Supporting Information).

Two isoforms (3 and 4) correspond to the soluble form of the receptor (Figure 2). InterPro analysis revealed regions consistent with complete immunoglobulin‐like and Type III fibronectin domains. DeepTMHMM identified the absence of the transmembrane region and the endodomain (Supporting Information).

Isoform 3 F3 corresponds to a complete intracellular peptide (Figure 2). InterPro analysis revealed the absence of immunoglobulin‐like and Type III fibronectin domains. DeepTMHMM confirmed that the ectodomain and the transmembrane region are absent. Isoform 5, in contrast, corresponds to a truncated intracellular peptide (Figure 2). InterPro and DeepTMHMM confirm findings similar to those for isoform 3 F3, with the main difference being the number of amino acids, as isoform 5 has a shorter sequence (Supporting Information).

Fourteen isoforms (7, 9, 14, 17, 18, 19, 21, 22, 23, 24, 25, 28, 29, 31) correspond to truncated peptides (Figure 2). Analysis with InterPro did not identify immunoglobulin‐like or Type III fibronectin domains, likely due to the very short sequence length. DeepTMHMM identified the absence of the transmembrane region and classified these residues as intracellular. However, alignment with CLUSTALW placed these residues within the ectodomain of the canonical protein, so these truncated peptides could be extracellular or intracellular (Supporting Information).

Two isoforms (2 F2 and 6) correspond to complete receptor structures with truncated extracellular domains (Figure 2). Analysis with InterPro revealed regions consistent with incomplete immunoglobulin‐like and Type III fibronectin domains, suggesting partial loss of extracellular domains D1–D3. DeepTMHMM identified the presence of the transmembrane region and endodomain (Supporting Information).

Four isoforms (12, 15, 20, and 30) correspond to complete receptor structures with truncated extracellular and intracellular domains (Figure 2). Analysis with InterPro revealed regions consistent with truncated immunoglobulin‐like and Type III fibronectin domains, suggesting partial loss of extracellular domains D1–D3. DeepTMHMM identified the complete transmembrane domain. Alignment with CLUSTALW placed some residues within the endodomain of the canonical protein (Supporting Information).

### 3.4 In Silico Characterization of IL‐17RA Isoforms

IL‐17RA consists of 866 amino acids; the signal peptide spans positions 1–32, the ectodomain 33–320, the transmembrane domain 321–341, and the endodomain 342–866 (www.dtu.biolib.com/DeepTMHMM). In the ectodomain, two type‐III fibronectin domains (D1 and D2) are present, while the SEFIR domain is located in the endodomain [37]. IL‐17RA isoform 2 (832 amino acids) corresponds to the soluble form of the receptor (Figure 2). Analysis with InterPro revealed regions consistent with complete Type III fibronectin domains. DeepTMHMM identified the transmembrane region as consisting of 10 amino acids, which is shorter than the canonical form of the receptor (21 amino acids). The endodomain is also present.

### 3.5 Expression of IL‐23R and IL‐17RA and Soluble Isoforms in Periodontitis

**FIGURE 3:**
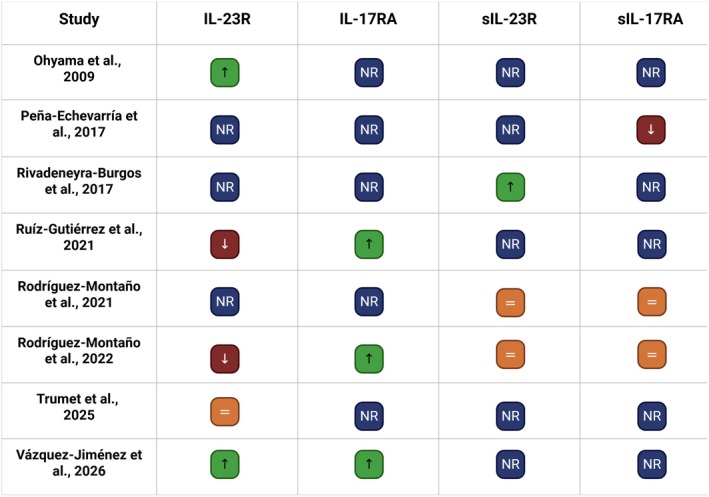
Effect direction plot of IL‐23R, IL‐17RA, sIL‐23R and sIL‐17RA expression in periodontitis. Each cell summarizes the direction of the finding reported by each study compared with periodontally healthy controls. Increased expression is represented by ↑, decreased expression by ↓, unchanged expression by =, and not reported outcomes by NR. Created in https://BioRender.com.

The clinical studies evaluating the expression of IL‐23R and IL‐17RA receptors included seven cross‐sectional articles and one pilot study. The earliest study was published in 2009, and the most recent in 2025. The studies were conducted in three countries: Mexico (75%), Japan (12.5%), and Germany (12.5%). A total of 287 participants (156 with periodontitis), with an average age of 42.4 years, were included. Various biofluids (serum, plasma, and gingival tissue) and techniques such as real‐time PCR, ELISA, immunofluorescence, and western blot were used. Scientific evidence has shown discrepancies in IL‐23R and IL‐17RA levels and their soluble isoforms (sIL‐23R and sIL‐17RA) across different biological samples and techniques. For the canonical forms of both receptors, Rodríguez‐Montaño et al., 2022 observed decreased plasma IL‐23R levels in patients with periodontitis compared to periodontally healthy individuals [33]. These findings are consistent with Ruiz‐Gutierrez et al., 2021, who reported decreased IL‐23R levels in gingival tissue from subjects with periodontitis [31]. Another study found no statistically significant differences in the proportion of cells expressing this receptor between tissues with severe periodontitis and healthy gingiva [34]. However, Ohyama et al. 2009 demonstrated increased *IL‐23R* mRNA expression in patients with periodontitis [28]. Similarly, our group recently found overexpression of IL‐23R using western blot in subjects with periodontitis compared to controls. In the same study, bands with different molecular weights were observed for both IL‐23R (50 and 100 kDa) and IL‐17RA (50, 100, and 200 kDa); however, the nature of these proteins was not further investigated (table 2 and Figure 3) [35].

Regarding the soluble isoform sIL‐23R, Rivadeneyra‐Burgos et al., 2016 demonstrated increased plasma levels of sIL‐23R in patients with periodontitis compared to periodontally healthy individuals [30]. Other studies found no statistically significant differences [32, 33]. Conversely, Ruiz‐Gutiérrez et al., 2021, Rodríguez‐Montaño et al., 2022, and Vázquez‐Jiménez et al., 2025 demonstrated increased IL‐17RA levels in plasma and gingival tissue in subjects with periodontitis compared to periodontally healthy individuals [31, 33, 35]. For the soluble isoform sIL‐17RA, Peña‐Echevarría et al., 2017 found increased serum and plasma levels in healthy subjects compared to patients with periodontitis [29], while Rodríguez‐Montaño et al., 2022 found no significant differences [33] (Table 2 and Figure 3).

**TABLE 2:** Expression of IL‐23R and IL‐17RA receptors and soluble isoforms (sIL‐23R and sIL‐17RA) in subjects with periodontitis.

Author and year	Country	Study design	Sex M/F	Average age	*N* health	*N* periodontitis	*N* total	Receptor	Biological sample/technique	Direction of change (↑/↓/NC) with statistical significance (*p*)
Ohyama et al. (2009)	Japan	Cross‐sectional	6/9	55.3	15	15	15	mRNA *IL23R*	Gingival tissue/real‐time PCR	↑ mRNA *IL23R* in periodontitis patients *p* = < 0.05
Peña‐Echeverría et al. (2017)	Mexico	Cross‐sectional	7/7	41.5	7	7	14	sIL‐17RA	Serum and plasma/ELISA	↑ sIL‐17RA in serum and plasma of healthy subjects *p* = 0.04
Rivadeneyra‐Burgos et al. (2017)	Mexico	Cross‐sectional	7/9	38.5	8	8	16	sIL‐23R	Serum and plasma/ELISA	↑ sIL‐23R in plasma of periodontitis patients *p* = 0.045
Ruíz‐Gutiérrez et al. (2021)	Mexico	Cross‐sectional	5/34	43	16	23	39	IL‐23R IL‐17RA	Gingival tissue/ELISA	↑ IL‐17RA in periodontitis patients *p* = 0.010 ↓ IL‐23R in periodontitis patients *p* = 0.034
Rodríguez‐Montaño et al. (2021)	Mexico	Cross‐sectional	Unclear	39.1	42	40	82	sIL‐23R sIL‐17RA	Plasma/ELISA	No significant differences between the two study groups *p* = ≥ 0.05
Rodríguez‐Montaño et al. (2022)	Mexico	Cross‐sectional	14/48	38.8	28	34	62	IL‐23R IL‐17RA sIL‐23R sIL‐17RA	Plasma/ELISA	↑ IL‐17RA in periodontitis patients *p* = ≥ 0.05 ↓ IL‐23R in periodontitis patients *p* = ≥ 0.05 sIL‐23 and sIL‐17RA showed no significant differences *p* = ≥ 0.05
Trumet et al. (2025)	Germany	Pilot	NR	NR	5	5	10	IL‐23R	Gingival tissue/Immunofluorescence	No significant differences between the two study groups *p* = 0.556
Vázquez‐Jiménez et al. (2026)	Mexico	Cross‐sectional	18/31	40.8	25	24	49	IL‐23R IL‐17RA	Gingival tissue/Western blot	↑ IL‐23R in periodontitis patients *p* = < 0.001 ↑ IL‐17RA in periodontitis patients *p* = 0.04

Abbreviations: ↑, Increased; ↓, Decreased; F, Female; IL‐17RA, Interleukin‐17 Receptor A; IL‐23R, Interleukin‐23 Receptor; M, male; NC, no change; NR, not reported; sIL‐17RA, soluble Interleukin‐17 Receptor A; sIL‐23R, soluble Interleukin‐23 Receptor.

### 3.6 Bias Outcomes

The studies included in this systematic review had a low risk of bias according to the QUIN and JBI assessment tools for cross‐sectional studies. For in vitro studies, five items—randomization, blinding, operator and evaluator details, and statistical analysis—were classified as not applicable because the included studies were mechanistic molecular biology research based on PCR, sequencing, and protein analysis. In this type of experimental study, these items are not relevant sources of bias, as the results are derived from highly standardized and descriptive methods (Table 3). Therefore, following methodological recommendations for evaluating molecular in vitro studies, a modified version of QUIN was applied, analysing only the criteria relevant to the experimental design of these studies. Most of the cross‐sectional studies met the eight JBI items, except for two studies [28, 34], which had methodological deficiencies in identifying and addressing confounding factors (Table 4).

**TABLE 3:** QUIN assessment of in vitro studies included.

QUIN (items)	Zhang et al. (2008)	Mancini et al. (2008)	Kan et al. (2008)	Sohda et al. (2013)
Clearly stated aims/objectives	2	2	2	2
Detailed explanation of sample size calculation	N/A	N/A	N/A	N/A
Detailed explanation of sampling technique	1	1	1	1
Details of comparison group	2	2	2	2
Detailed explanation of methodology	2	2	2	2
Operator details	N/A	N/A	N/A	N/A
Randomization	N/A	N/A	N/A	N/A
Method of measurement of Outcome	2	2	2	2
Outcome assessor details	N/A	N/A	N/A	N/A
Blinding	N/A	N/A	N/A	N/A
Statistical analysis	N/A	N/A	N/A	N/A
Results	2	2	2	2
Final score	11 = 78.6%	11 = 78.6%	11 = 78.6%	11 = 78.6%
Bias	Low	Low	Low	Low

*Note:* Green means low bias.

**TABLE 4:** JBI assessment of cross‐sectional studies included.

JBI (items)	Ohyama et al. (2009)	Peña‐Echeverría et al. (2017)	Rivadeneyra‐Burgos et al. (2017)	Ruíz‐Gutiérrez et al. (2021)	Rodríguez‐Montaño et al. (2021)	Rodríguez‐Montaño et al. (2022)	Trumet et al. (2025)	Vázquez‐Jiménez et al. (2025)
Were the criteria for inclusion in the sample clearly defined?	Yes	Yes	Yes	Yes	Yes	Yes	Yes	Yes
Were the study subjects and the setting described in detail?	Yes	Yes	Yes	Yes	Yes	Yes	Yes	Yes
Was the exposure measured in a valid and reliable way?	Yes	Yes	Yes	Yes	Yes	Yes	Yes	Yes
Were objective, standard criteria used for measurement of the condition?	Yes	Yes	Yes	Yes	Yes	Yes	Yes	Yes
Were confounding factors identified?	Unclear	Yes	Yes	Yes	Yes	Yes	Unclear	Yes
Were strategies to deal with confounding factors stated?	No	Yes	Yes	Yes	Yes	Yes	No	Yes
Were the outcomes measured in a valid and reliable way?	Yes	Yes	Yes	Yes	Yes	Yes	Yes	Yes
Was appropriate statistical analysis used?	Yes	Yes	Yes	Yes	Yes	Yes	Yes	Yes
Final score	75%	100%	100%	100%	100%	100%	75%	100%
Bias	Low	Low	Low	Low	Low	Low	Low	Low

*Note:* Green means low bias.

## 4 Discussion

This systematic review is the first comprehensive synthesis of the available evidence on splicing variants and isoforms of the IL‐23R and IL‐17RA receptors, as well as their possible relationship with periodontitis. One of the most relevant findings is the remarkable diversity of splicing variants described for the *IL23R* gene, which produces multiple isoforms with structural differences. Since the initial studies by Zhang et al., 2006, Mancini et al., 2008 and Kan et al., 2008, it has been documented that *IL23R* can generate numerous transcripts through partial or complete exon deletions by alternative splicing, giving rise to soluble isoforms and structurally distinct receptor variants, including proteins with truncated extracellular and/or intracellular domains [13, 14, 15]. Our multiple alignment analysis confirms that these variants are not simple transcriptional irregularities, but can be translated into proteins with different structures, suggesting potentially different biological functions.

From a functional perspective, the generation of IL‐23R isoforms with truncated extracellular or intracellular domains could significantly alter the classic JAK/STAT signalling pathway, as observed with certain missense variants of IL‐23R (G149R, V362I, and R381Q) in inflammatory bowel diseases, where single amino acid changes occur in the extracellular, transmembrane, and intracellular domains. These variants result in a loss‐of‐function (protective) phenotype, characterized by lower IL‐23R expression on the cell surface and reduced STAT3 and STAT4 phosphorylation after IL‐23 stimulation [38].

It has also been shown that soluble isoforms of IL‐23R can be generated by cell membrane cleavage by adamalysins, mainly ADAM10 and ADAM17, which act as negative regulators by sequestering their ligand. This modulates the intensity of the immune response [39].

Similarly, the presence of multiple IL‐23R isoforms within the same inflamed tissue could affect the stability of the Th17 phenotype. IL‐23 is essential for the clonal expansion and maintenance of these cells [7]. Any alteration in the availability or functionality of its receptor could indirectly modulate the production of IL‐17, IL‐22, and RANKL, which are key molecules in alveolar bone destruction. Therefore, rather than viewing IL‐23R as a single entity, scientific evidence indicates that it should be understood as a dynamic set of isoforms whose relative proportions may vary depending on the inflammatory microenvironment, disease stage, and cell type.

In contrast to the complexity observed in the *IL23R* gene, the *IL17RA* gene exhibits much more limited splicing diversity. Sodha et al. experimentally demonstrated that sIL‐17RA is produced by a complete deletion of exon 11 (pΔ11), which encodes the transmembrane region, and also detected this protein in the culture media of human cell lines using Western blot [16].

Multiple sequence alignment analysis with CLUSTALW confirmed sequence differences with partial deletions compared to the canonical 866‐amino‐acid form, particularly in the protein's transmembrane region. InterPro showed that this isoform retains important domains, including the fibronectin‐like extracellular domains III (IL17R_fnIII_D1/D2) and the intracellular SEFIR domain, indicating that the structural architecture of the receptor is partially preserved. DeepTMHMM revealed a shorter hydrophobic region (transmembrane domain–10 amino acids) compared to the canonical form of the receptor (21 amino acids), suggesting that, although residual hydrophobic residues remain, the reduced length of this segment is likely insufficient to maintain stable membrane anchoring, resulting in sIL‐17RA.

However, this apparent simplicity does not diminish its biological importance or the possibility of discovering additional splicing variants. IL‐17RA is involved in the activation of proinflammatory pathways (TRAF6/TAK1/NF‐κB) [40], which are crucial to amplifying the inflammatory response in periodontitis [9].

The presence of sIL‐17RA presents an interesting regulatory scenario. Increased levels of this isoform have been demonstrated in periodontally healthy subjects compared to patients with periodontitis [27], while other studies have found no statistically significant differences [32, 33]. These discrepancies could be explained, in part, by the possible role of sIL‐17RA as a decoy receptor, capable of limiting the availability of IL‐17A and potentially attenuating its proosteoclastogenic effect [16]. It is also plausible that the observed differences are due to variations in disease stage, the biological source analysed, or the affinity and specificity of the antibodies used in immunoenzymatic assays. In gingival tissue, increased expression of IL‐17RA suggests that local IL‐17 signalling remains active in periodontitis, promoting RANKL expression and osteoclastogenesis [29, 33]. However, the possible coexistence of the canonical form with the soluble isoform could modulate this signalling, reinforcing the idea that the balance between isoforms, rather than the absolute expression of the receptor, may be a key determinant of the tissue response.

An important aspect of this review is the methodological heterogeneity among the included clinical studies. The available clinical evidence is limited and exploratory, and does not permit causal inferences. All included clinical studies were cross‐sectional, preventing assessment of temporal relationships between receptor expression patterns and periodontal disease progression. The total number of participants across clinical studies was modest (*n* = 287), which limits statistical robustness and external validity. Therefore, findings should be interpreted as hypothesis‐generating rather than as definitive evidence of a pathogenic role. The predominance of studies from a single research group may limit geographical generalizability and underscores the need for independent replication in diverse populations.

Differences in the type of biological sample (serum, plasma, or gingival tissue), the analytical technique used (real‐time PCR, ELISA, immunofluorescence, or Western blot), and the study design may significantly contribute to the discordant results observed. For example, while PCR quantifies messenger RNA levels without indicating whether they translate into functional proteins, techniques such as Western blot detect multiple protein isoforms, revealing bands of different molecular weights that may go unnoticed in immunoenzymatic assays.

Most antibodies used in ELISA are designed against specific epitopes of the canonical receptor, which may limit the detection of truncated or soluble isoforms. This technical bias is particularly relevant for IL‐23R, where structural diversity is extensive. Consequently, the discrepancies reported between studies do not necessarily reflect true biological contradictions, but rather different observation windows of a complex molecular system.

The identification of multiple IL‐23R isoforms and a functional soluble isoform of IL‐17RA provides new insights into the pathogenesis of periodontitis. These isoforms may modulate inflammatory signalling and could serve as more specific diagnostic or prognostic biomarkers than measurements of individual cytokines. Clinical experience with IL‐17RA receptor antagonists in diseases such as psoriasis also demonstrates that receptors, not only ligands, are viable therapeutic targets [41].

To further clarify the role of the IL‐23/IL‐17 axis in periodontitis, future studies could include: (1) multi‐omics analyses integrating transcriptomics, proteomics, and single‐cell RNA sequencing to comprehensively profile IL‐23R and IL‐17RA isoform expression across disease stages in gingival tissues and peripheral blood from periodontitis patients and healthy controls, addressing methodological heterogeneity; (2) functional assays using CRISPR‐edited cell lines or primary Th17 cells to evaluate how specific IL‐23R isoforms alter JAK/STAT signalling, Th17 differentiation, and downstream cytokine production (such as IL‐17A, IL‐22, and RANKL); (3) longitudinal cohort studies assessing dynamic changes in sIL‐17RA and sIL‐23R levels in serum and saliva during periodontitis progression and treatment, correlating these with clinical parameters such as alveolar bone loss; (4) in vivo models, such as ligature‐induced periodontitis in IL‐23R isoform‐specific knockout mice, to determine causal contributions to osteoclastogenesis and tissue destruction; and (5) validation of isoform‐specific antibodies for ELISA and Western blotting to improve detection accuracy and explore their utility as prognostic biomarkers. Rather than proposing immediate clinical applications, this review highlights a conceptual gap between molecular receptor diversity and periodontal immunopathology that warrants targeted experimental investigation. Bridging this gap will require mechanistic validation and well‐designed longitudinal clinical studies before translational implications can be established.

## 5 Conclusion

This systematic review demonstrates that IL‐23R has multiple splicing variants, resulting in different transcripts and protein isoforms. Bioinformatic analysis indicates that some of these isoforms lack domains required for ligand binding, membrane anchoring, or intracellular signalling, or contain truncated versions of these domains, suggesting a potential impact in the regulation of IL‐23R‐mediated JAK/STAT signalling and Th17 cell‐related responses. However, these functional consequences remain hypothetical and require experimental validation. In contrast, IL‐17RA exhibits more limited alternative splicing, with the soluble isoform (sIL‐17RA) described in the literature as a potential decoy receptor. Nevertheless, its anti‐inflammatory role in periodontitis was not directly demonstrated by the clinical studies included in this review. Therefore, current evidence supports the presence of structural diversity among isoforms, while the functional relevance of these isoforms in periodontitis remains a hypothesis for future mechanistic studies.

## Author Contributions

**Mario Alberto Alarcón‐Sánchez:** conceptualization, investigation, writing – original draft, methodology, validation, visualization, writing – review and editing, software, formal analysis, project administration, data curation, supervision, resources. **Celia Guerrero‐Velázquez:** visualization, validation, supervision, formal analysis. **Javier Flores‐Fraile:** writing – review and editing, writing – original draft, visualization, validation, supervision, resources, project administration, investigation, formal analysis, data curation, conceptualization. **Daniel Ortuño‐Sahagún:** writing – review and editing, writing – original draft, visualization, validation, supervision, formal analysis. **Sarah Monserrat Lomelí‐Martínez:** writing – review and editing, writing – original draft, visualization, validation, supervision, methodology, formal analysis, conceptualization. **Artak Heboyan:** writing – review and editing, writing – original draft, visualization, validation, supervision, resources, project administration, investigation, formal analysis, data curation, conceptualization. **Julieta Sarai Becerra‐Ruiz:** writing – review and editing, writing – original draft, visualization, validation, supervision, methodology, formal analysis, conceptualization.

## Funding

The authors have nothing to report.

## Ethics Statement

The authors have nothing to report.

## Consent

The authors have nothing to report.

## Conflicts of Interest

The authors declare no conflicts of interest.

## Supporting information


**Data S1:** Supplementary Material.

## Data Availability

The data that support the findings of this study are available from the corresponding author upon reasonable request.
