# Supplementary material for: Interleukin‐23 Receptor and Interleukin‐17 Receptor A: Splice Variants, Isoforms and Their Relationship With Periodontitis—A Systematic Review and Bioinformatic Analysis
Source: J Cell Mol Med. 2026 Aug 17;30(16):e71325. doi: 10.1111/jcmm.71325 (PMC13482007; doi:10.1111/jcmm.71325)
Supplement: Supplementary file 1 [file JCMM-30-e71325-s001.docx]

**Supplementary Material**

**A**

**B**

Analysis using InterPro and DeepTMHMM for the canonical form of IL-23R (629 amino acids). A) InterPro shows conserved structural domains: Immunoglobulins and Fibronectin type III; B) Topological prediction using DeepTMHMM identified that the transmembrane region consists of 21 amino acids. Based on the reported values, the model has a high confidence level in the prediction.

**IL-23R**

Isoform 2- F1

**A**

**B**

Analysis with InterPro and DeepTMHMM for the IL-23R isoform 2F1 (138 amino acids). A) InterPro shows part of the Immunoglobulins domain; B) Topological prediction using DeepTMHMM identified the absence of the receptor's transmembrane region and endodomain. Based on the reported values, the model has high confidence in its prediction.

Isoform 2- F2

**A**

**B**

Analysis with InterPro and DeepTMHMM for the IL-23R isoform 2F2 (375 amino acids). A) InterPro shows part of the Immunoglobulins and Fibronectin type III domain truncated; B) Topological prediction using DeepTMHMM identified that the transmembrane region consists of 21 amino acids (complete). Based on the reported values, the model has high confidence in the prediction.

Isoform 3- F1

**A**

**B**

Analysis with InterPro and DeepTMHMM for the IL-23R isoform 3F1 (320 amino acids). InterPro shows conserved structural domains of Immunoglobulins and Fibronectin type III; B) Topological prediction using DeepTMHMM identified the absence of the receptor's transmembrane region and endodomain. Based on the reported values, the model has high confidence in its prediction.

Isoform 3- F3

**A**

**B**

Analysis with InterPro and DeepTMHMM for the IL-23R isoform 3F3 (264 amino acids). InterPro shows the Immunoglobulins and Fibronectin type III domains are absent; B) Topological prediction using DeepTMHMM identified the absence of the ectodomain and the transmembrane region of the receptor. Based on the reported values, the model has high confidence in the prediction.

Isoform 4

**A**

**B**

Analysis with InterPro and DeepTMHMM for the IL-23R isoform 4 (320 amino acids). InterPro shows conserved structural domains of Immunoglobulins and Fibronectin type III; B) Topological prediction using DeepTMHMM identified the absence of the receptor's transmembrane region and endodomain. Based on the reported values, the model has high confidence in its prediction.

Isoform 5

**A**

**B**

Analysis with InterPro and DeepTMHMM for the IL-23R isoform 5 (227 amino acids). InterPro shows the Immunoglobulins and Fibronectin type III domains are absent; B) Topological prediction using DeepTMHMM identified the absence of the ectodomain and the transmembrane region of the receptor. Based on the reported values, the model has high confidence in the prediction.

Isoform 6

**A**

**B**

Analysis with InterPro and DeepTMHMM for the IL-23R isoform 6 (391 amino acids). A) InterPro shows part of the Immunoglobulins domain truncated; B) Topological prediction using DeepTMHMM identified that the transmembrane region consists of 21 amino acids (complete). Based on the reported values, the model has high confidence in the prediction.

Isoform 7

**A**

**B**

Analysis using InterPro and DeepTMHMM for IL-23R isoform 7 (45 amino acids). InterPro shows that the Immunoglobulins and Fibronectin type III domains are absent; B) Topological prediction using DeepTMHMM identified the absence of the receptor’s ectodomain and transmembrane region. Based on the reported values, the model has a high confidence in the prediction.

Note: CLUSTALW shows residues belonging to the ectodomain, while DeepTMHMM shows residues belonging to the endodomain.

Isoform 8

**A**

**B**

Analysis with InterPro and DeepTMHMM for the IL-23R isoform 8 (88 amino acids). InterPro shows the Immunoglobulins and Fibronectin type III domains are absent; B) Topological prediction using DeepTMHMM identified the absence of the receptor's transmembrane region and endodomain. Based on the reported values, the model has high confidence in its prediction.

Isoform 9

**A**

**B**

Analysis with InterPro and DeepTMHMM for the IL-23R isoform 9 (28 amino acids). InterPro shows the Immunoglobulins and Fibronectin type III domains as absent; B) Topological prediction using DeepTMHMM identified the absence of the ectodomain and the receptor's transmembrane region. Based on the reported values, the model has high confidence in its prediction.

Note: CLUSTALW shows residues belonging to the ectodomain, while DeepTMHMM shows residues belonging to the endodomain.

Isoform 10

**A**

**B**

Analysis with InterPro and DeepTMHMM for the IL-23R isoform 10 (72 amino acids). InterPro shows the Immunoglobulins and Fibronectin type III domains as truncated; B) Topological prediction using DeepTMHMM identified the absence of the transmembrane region and the receptor endodomain. Based on the reported values, the model has high confidence in its prediction.

Isoform 11

**A**

**B**

Analysis with InterPro and DeepTMHMM for the IL-23R isoform 11 (137 amino acids). InterPro shows the Immunoglobulins and Fibronectin type III domains as truncated; B) Topological prediction using DeepTMHMM identified the absence of the transmembrane region and the receptor endodomain. Based on the reported values, the model has high confidence in its prediction.

Isoform 12

**A**

**B**

Analysis with InterPro and DeepTMHMM for the IL-23R isoform 12 (270 amino acids). A) InterPro shows part of the Immunoglobulins and Fibronectin type III domains truncated; B) Topological prediction using DeepTMHMM identified that the transmembrane region consists of 21 amino acids (complete). Based on the reported values, the model has high confidence in the prediction.

Isoform 13

C

**A**

**B**

Analysis with InterPro and DeepTMHMM for the IL-23R isoform 13 (210 amino acids). InterPro shows truncated Immunoglobulins and Fibronectin type III domains; B) Topological prediction using DeepTMHMM identified the absence of the receptor's transmembrane region and endodomain. Based on the reported values, the model has high confidence in its prediction.

Isoform 14

**A**

**B**

Analysis with InterPro and DeepTMHMM for the IL-23R isoform 14 (45 amino acids). InterPro shows the Immunoglobulins and Fibronectin type III domains as absent; B) Topological prediction using DeepTMHMM identified the absence of the ectodomain and the receptor's transmembrane region. Based on the reported values, the model has high confidence in its prediction.

Note: CLUSTALW shows residues belonging to the ectodomain, while DeepTMHMM shows residues belonging to the endodomain.

Isoform 15

**A**

**B**

Analysis with InterPro and DeepTMHMM for the IL-23R isoform 15 (199 amino acids). A) InterPro shows part of the Immunoglobulins and Fibronectin type III domain truncated; B) Topological prediction using DeepTMHMM identified that the transmembrane region consists of 21 amino acids (complete). Based on the reported values, the model has high confidence in the prediction.

Isoform 16

**A**

**B**

Analysis with InterPro and DeepTMHMM for the IL-23R isoform 16 (180 amino acids). InterPro shows the Immunoglobulins and Fibronectin type III domains as truncated; B) Topological prediction using DeepTMHMM identified the absence of the receptor's transmembrane region and endodomain. Based on the reported values, the model has high confidence in its prediction.

Isoform 17

**A**

**B**

Analysis with InterPro and DeepTMHMM for the IL-23R isoform 17 (28 amino acids). InterPro shows the Immunoglobulins and Fibronectin type III domains as absent; B) Topological prediction using DeepTMHMM identified the absence of the ectodomain and the transmembrane region of the receptor. Based on the reported values, the model has high confidence in its prediction.

Note: CLUSTALW shows residues belonging to the ectodomain of the canonical receptor form, while DeepTMHMM shows residues belonging to the endodomain.

Isoform 18

**A**

**B**

Analysis with InterPro and DeepTMHMM for the IL-23R isoform 18 (31 amino acids). InterPro shows the Immunoglobulins and Fibronectin type III domains as absent; B) Topological prediction using DeepTMHMM identified the absence of the ectodomain and the transmembrane region of the receptor. Based on the reported values, the model has high confidence in its prediction.

Note: CLUSTALW shows residues belonging to the ectodomain of the canonical receptor form, while DeepTMHMM shows residues belonging to the endodomain.

Isoform 19

**A**

**B**

Analysis using InterPro and DeepTMHMM for IL-23R isoform 19 (45 amino acids). InterPro shows that the Immunoglobulins and Fibronectin type III domains are absent; B) Topological prediction using DeepTMHMM identified the absence of the receptor’s ectodomain and transmembrane region. Based on the reported values, the model has a high confidence in the prediction.

Note: CLUSTALW shows residues belonging to the ectodomain, while DeepTMHMM shows residues belonging to the endodomain.

Isoform 20

Analysis with InterPro and DeepTMHMM for the IL-23R isoform 20 (268 amino acids). A) InterPro shows part of the Immunoglobulins and Fibronectin type III domains truncated; B) Topological prediction using DeepTMHMM identified that the transmembrane region consists of 21 amino acids (complete). Based on the reported values, the model has high confidence in the prediction.

Isoform 21

Analysis with InterPro and DeepTMHMM for the IL-23R isoform 21 (39 amino acids). InterPro shows the Immunoglobulins and Fibronectin type III domains as absent; B) Topological prediction using DeepTMHMM identified the absence of the ectodomain and the transmembrane region of the receptor. Based on the reported values, the model has high confidence in its prediction.

Note: CLUSTALW shows residues belonging to the ectodomain of the canonical receptor form, while DeepTMHMM shows residues belonging to the endodomain.

Isoform 22

**A**

**B**

Analysis with InterPro and DeepTMHMM for the IL-23R isoform 22 (28 amino acids). InterPro shows the Immunoglobulins and Fibronectin type III domains as absent; B) Topological prediction using DeepTMHMM identified the absence of the ectodomain and the receptor's transmembrane region. Based on the reported values, the model has high confidence in its prediction.

Note: CLUSTALW shows residues belonging to the ectodomain, while DeepTMHMM shows residues belonging to the endodomain.

Isoform 23

**A**

**B**

Analysis with InterPro and DeepTMHMM for the IL-23R isoform 23 (28 amino acids). InterPro shows the Immunoglobulins and Fibronectin type III domains as absent; B) Topological prediction using DeepTMHMM identified the absence of the ectodomain and the receptor's transmembrane region. Based on the reported values, the model has high confidence in its prediction.

Note: CLUSTALW shows residues belonging to the ectodomain, while DeepTMHMM shows residues belonging to the endodomain.

Isoform 24

**A**

**B**

Analysis with InterPro and DeepTMHMM for the IL-23R isoform 24 (45 amino acids). InterPro shows the Immunoglobulins and Fibronectin type III domains as absent; B) Topological prediction using DeepTMHMM identified the absence of the ectodomain and the receptor's transmembrane region. Based on the reported values, the model has high confidence in its prediction.

Note: CLUSTALW shows residues belonging to the ectodomain, while DeepTMHMM shows residues belonging to the endodomain.

Isoform 25

**A**

**B**

Analysis with InterPro and DeepTMHMM for the IL-23R isoform 25 (45 amino acids). InterPro shows the Immunoglobulins and Fibronectin type III domains as absent; B) Topological prediction using DeepTMHMM identified the absence of the ectodomain and the receptor's transmembrane region. Based on the reported values, the model has high confidence in its prediction.

Note: CLUSTALW shows residues belonging to the ectodomain, while DeepTMHMM shows residues belonging to the endodomain.

Isoform 26

**A**

**B**

Analysis with InterPro and DeepTMHMM for the IL-23R isoform 26 (147 amino acids). InterPro shows the Immunoglobulins and Fibronectin type III domains as truncated; B) Topological prediction using DeepTMHMM identified the absence of the receptor's transmembrane region and endodomain. Based on the reported values, the model has high confidence in its prediction.

Isoform 27

**A**

**B**

Analysis with InterPro and DeepTMHMM for the IL-23R isoform 27 (112 amino acids). InterPro shows the Immunoglobulins and Fibronectin type III domains as truncated; B) Topological prediction using DeepTMHMM identified the absence of the receptor's transmembrane region and endodomain. Based on the reported values, the model has high confidence in its prediction.

Isoform 28

**A**

**B**

Analysis with InterPro and DeepTMHMM for the IL-23R isoform 28 (31 amino acids). InterPro shows the Immunoglobulins and Fibronectin type III domains as absent; B) Topological prediction using DeepTMHMM identified the absence of the ectodomain and the transmembrane region of the receptor. Based on the reported values, the model has high confidence in its prediction.

Note: CLUSTALW shows residues belonging to the ectodomain of the canonical receptor form, while DeepTMHMM shows residues belonging to the endodomain.

Isoform 29

**A**

**B**

Analysis using InterPro and DeepTMHMM for IL-23R isoform 29 (45 amino acids). InterPro shows that the Immunoglobulins and Fibronectin type III domains are absent; B) Topological prediction using DeepTMHMM identified the absence of the receptor’s ectodomain and transmembrane region. Based on the reported values, the model has a high confidence in the prediction.

Note: CLUSTALW shows residues belonging to the ectodomain, while DeepTMHMM shows residues belonging to the endodomain.

Isoform 30

**A**

**B**

Analysis with InterPro and DeepTMHMM for the IL-23R isoform 30 (238 amino acids). A) InterPro shows part of the Immunoglobulin and Fibronectin type III domains truncated; B) Topological prediction using DeepTMHMM identified that the transmembrane region consists of 21 amino acids (complete). Based on the reported values, the model has high confidence in the prediction.

Isoform 31

**A**

**B**

Analysis with InterPro and DeepTMHMM for the IL-23R isoform 31 (31 amino acids). InterPro shows the Immunoglobulins and Fibronectin type III domains as absent; B) Topological prediction using DeepTMHMM identified the absence of the ectodomain and the transmembrane region of the receptor. Based on the reported values, the model has high confidence in its prediction.

Note: CLUSTALW shows residues belonging to the ectodomain of the canonical receptor form, while DeepTMHMM shows residues belonging to the endodomain.

IL-17RA - Isoform 2

**A**

**B**

Analysis with InterPro and DeepTMHMM for the canonical form of IL-17RA (866 amino acids). A) InterPro shows conserved structural domains IL17R_fnIII_D1/D2 and SEFIR19; B) Topological prediction using DeepTMHMM identified that the transmembrane region consists of 21 amino acids. Based on the reported values, the model has high confidence in the prediction.

**A**

Analysis with InterPro and DeepTMHMM for the IL-17RA isoform 2 (832 amino acids). A) InterPro shows conserved structural domains IL17R_fnIII_D1/D2 and SEFIR19; B) Topological prediction using DeepTMHMM identified a shorter hydrophobic segment (10 amino acids) compared to the canonical receptor form (21 amino acids). Based on the reported values, the model has high confidence in the prediction.

**B**

**Considerations for CLUSTALW analysis:**

The orange box indicates the amino acids that make up the signal peptide, the blue box indicates the amino acids that make up the extracellular domain, the green box indicates the amino acids that make up the transmembrane domain, and the purple box indicates the amino acids that make up the intracellular domain.

The isoforms highlighted in different colors correspond to the same amino acid sequences but are generated from different splicing variants.
